# Exploring hub genes related to adipocytokines in keloids: a combined analysis integrating single-cell, Mendelian randomization and bulk transcriptome data with experimental verification

**DOI:** 10.3389/fmolb.2026.1740876

**Published:** 2026-03-11

**Authors:** Maisude Mahemuti, Youyou Cheng, Minxuan Li, Alimire Yilihamu

**Affiliations:** Department of Plastic Surgery, People’s Hospital of Xinjiang Uygur Autonomous Region, Urumqi, China

**Keywords:** adipocytokine, hub genes, keloids, mendelian randomization analysis, single-cellanalysis

## Abstract

**Background:**

Keloids are fibroproliferative skin tumors with excessive collagen deposition and are strongly linked to adipocytokine dysregulation. However, the underlying mechanisms remain unclear. This study aimed to identify potential therapeutic targets and elucidate adipocytokine-related pathological mechanisms underlying keloid formation.

**Methods:**

Hub genes were identified using differential expression analysis and machine learning. Mendelian randomization assessed causal relationships. Functional insights were gained through gene set enrichment analysis (GSEA) and drug prediction. Single-cell RNA sequencing (scRNA-seq) identified key cell types, and RT-qPCR validated gene expression.

**Results:**

We identified 818 differentially expressed genes, narrowing to seven key genes and two hub genes: PIK3R3 and ANGPTL5. MR indicated PIK3R3 as a causal risk factor for keloids, while ANGPTL5 showed no causal association. GSEA linked PIK3R3 to the TEL pathway and ANGPTL5 to adipocyte differentiation. Drug predictions included harmine for PIK3R3 and silica for ANGPTL5. scRNA-seq highlighted fibroblasts as key cells expressing these genes. RT-qPCR confirmed PIK3R3 upregulation in keloids, though ANGPTL5 results were inconsistent, possibly due to sample limitations.

**Conclusion:**

This study, based on the integrated re-analysis of existing publicly available transcriptomic data and combined with clinical sample validation, revealed the potential hub roles of PIK3R3 and ANGPTL5 in keloid pathogenesis. PIK3R3 was validated as a causal hub gene, while ANGPTL5 also showed relevance. The study provides new molecular evidence and mechanistic insights for understanding the adipocytokine-related pathological mechanisms of keloids, and suggests potential therapeutic directions such as harmine for future research.

## 1 Introduction

Keloids are pathological scars with an incompletely understood pathogenesis. Genetic predisposition, inflammatory responses, and fibroblast dysfunction are implicated, leading to excessive fibroblast proliferation and extracellular matrix (ECM) deposition. Keloids cause cosmetic concerns, pain, and functional impairment (He and Song, 2025). They frequently develop in high-tension areas like the chest and shoulders, with higher prevalence in individuals with darker skin (Xiao et al., 2024). Multimodal therapy is essential for reducing recurrence. Core treatments include intralesional corticosteroids and surgical excision combined with postoperative radiotherapy or drug injections. Adjuvant therapies like laser, pressure garments, and silicone gels require long-term use (Wu et al., 2025). Due to high recurrence rates, identifying novel molecular mechanisms and therapeutic targets is crucial for improving patient prognosis and quality of life (Xie R. et al., 2024).

Adipocytokines, secreted by adipose tissue, significantly influence cancer development and progression. They modulate energy metabolism and inflammation, impacting cancer through effects on the tumor microenvironment, metastasis promotion, and cachexia (Fan et al., 2024; Liu and Li, 2025). These factors are also closely linked to keloid formation, regulating inflammation, promoting fibrosis, and altering ECM metabolism. Pro-inflammatory adipocytokines like leptin, IL-6, and TNF-α activate keloid fibroblasts, causing abnormal collagen accumulation. Resistin enhances fibrosis via the TGF-β/Smad pathway, while reduced adiponectin weakens anti-inflammatory and anti-fibrotic effects, collectively driving keloid proliferation and persistence (Darmawan et al., 2020). Chronic inflammation and metabolic imbalance mediated by adipocytokines are key mechanisms hindering keloid regression, making exploration of their molecular mechanisms vital for improved therapies.

Mendelian randomization (MR), an epidemiological method, utilizes genetic variants—among which single nucleotide polymorphisms (SNPs) are a common type—as instrumental variables. The aim of this technique is to infer causal relationships between exposures (e.g., gene expression levels, lifestyle) and outcomes related to health (e.g., disease risk) (Qi et al., 2025). Three core assumptions underpin the application of MR: 1) the instrumental variable and the exposure are strongly associated (assumption of “relevance”); 2) the instrumental variable has no correlation with confounding factors (assumption of “independence”); 3) the instrumental variable’s impact on the outcome is mediated solely through the exposure, without additional pathways (assumption of “exclusion restriction”) (Luo et al., 2025). MR helps overcome limitations of observational studies and clarify causal direction. For example, MR identified CCND2 as a risk factor and KLF4 as protective against keloids (He and Song, 2025).

Leveraging multiple transcriptomic datasets from public databases, this study aims to identify adipocytokine-related hub genes in keloids through a systematic re-analysis. By integrating differential expression analysis with machine learning algorithms, we seek to elucidate the roles of these genes in fibrosis and inflammation and evaluate their potential as therapeutic targets. Subsequently, MR analysis is employed to investigate the causal relationships between these hub genes and keloid formation, clarifying their regulatory mechanisms in disease pathogenesis. Furthermore, single-sell analysis characterizes the expression patterns of these genes within key cell subpopulations to uncover cell-type-specific regulatory networks. Finally, the consistency between bioinformatic predictions and clinical reality is rigorously validated via RT-qPCR in clinical samples to ensure the reliability of the findings.

## 2 Materials and methods

### 2.1 Data acquirement

The Gene Expression Omnibus (GEO) database (https://www.ncbi.nlm.nih.gov/geo/) served as the source for acquiring transcriptome datasets of keloids. The GSE113619 (GPL21290 and GPL16791) consists of the expression profiles of 18 keloid patients’ (keloid) and 14 healthy control skin’ (normal) tissue samples (Samples other than those of 42 days were excluded). Additionally, the GSE158395 (GPL24676) consists of the expression profiles of four keloid patients’ (keloid) and six healthy control skin’ (normal) tissue samples (Wu et al., 2020) (Three non-lesional skins of keloid patients were removed). Comprising expression profiles of tissue samples from three keloid patients (keloid tissues) and three normal scar samples (normal tissues), the single-cell RNA sequencing (scRNA-seq) dataset was GSE163973 (using the GPL24676 platform) (Deng et al., 2021). Transcriptomic data preprocessing primarily consisted of two steps: firstly, raw count data for GSE113619 and GSE158395 were obtained from the GEO database, gene IDs were uniformly converted to standard gene symbols, and keloid lesion samples along with matched normal control samples were selected and retained; subsequently, gene effective lengths were calculated based on the GENCODE v36 annotation file, raw counts were converted into Transcripts Per Million (TPM) values, and a final log2 (TPM+1) transformation was applied to meet the distribution requirements for downstream analyses. Regarding batch effect assessment, both datasets were derived from independent experiments with relatively small sample sizes, and no explicit batch correction was performed. The main strategies for controlling potential batch influences were the application of TPM normalization to mitigate sequencing depth and gene length biases, and conducting independent analyses for each dataset to avoid direct cross-dataset comparisons.

Moreover, 368 adipocytokine-related genes (ARGs) in total were obtained from relevant literature; these genes are listed in Supplementary Table 1 (Fan et al., 2024). We retrieved the genetic variation data for keloids (identifier: ebi-a-GCST90018874) from the Integrative Epidemiology Unit Open genome-wide association studies (IEU OpenGWAS) website at https://gwas.mrcieu.ac.uk/. The dataset comprised 481,912 patients of European ancestry (ncase = 668; ncontrol = 481,244) and 24,197,210 single nucleotide polymorphisms (SNPs) in total. Through IEU OpenGWAS, we obtained expression quantitative trait locus (eQTL) data for the genes associated with exposure factors; this involved converting genes to Ensemble Identification (IDs) to generate exposure IDs. All data were downloaded on 7 April 2025.

### 2.2 Identification of differentially expressed genes (DEGs)

To compare keloid samples and normal samples in GSE113619, differential expression analysis was carried out with the DESeq2 package (version 1.40.2) (Zhang X. et al., 2024). The identification of DEGs was based on two threshold criteria: |log2Fold Change (FC)| greater than 0.5 and p-values less than 0.05. The ggplot2 package (v 3.5.1) served to visualize the differential expression results (Xie Z. W. et al., 2024). Thereafter, the pheatmap package (v 1.0.12) was employed to produce a heatmap. This heatmap included the top 10 upregulated and top 10 downregulated genes, selected according to the largest |log2FC| values (Sun et al., 2022).

### 2.3 Identification and function analyses of candidate genes

By leveraging the ggvenn package (v 0.1.10), the overlap between DEGs and ARGs was examined to identify candidate genes (Schaarschmidt et al., 2021). The biological pathways and functional mechanisms of candidate genes in keloid were examined to understand their roles and interactions. The clusterProfiler package (version 4.15.0.3) was applied to carry out two types of enrichment analysis for the candidate genes: Gene Ontology (Suzuki et al., 2025) enrichment analysis (with adjusted p-values [p.adjust] < 0.05) and Kyoto Encyclopedia of Genes and Genomes (KEGG) pathway enrichment analysis (p.adjust <0.05) (Huang et al., 2024). The GO analysis mainly encompasses three parts, specifically biological process (BP), molecular function (MF), and cellular component (CC). We employed the GOplot package (version 1.0.2) (Dang et al., 2022) to generate visualizations of the top 10 enriched pathways obtained from both GO and KEGG. The determination of these pathways relied on the minimum p. adjust-values. Meanwhile, via the STRING database (Search Tool for the Retrieval of Interacting Genes/Proteins, accessible at https://string-db.org/), a protein-protein interaction (PPI) network was constructed for the candidate genes, using the criterion that the overall score must be ≥0.4. Isolated gene nodes without PPI with other genes were removed to draw the network diagram.

### 2.4 Identification of hub genes

To screen for key genes in the GSE113619 dataset, three different machine learning algorithms were used. To elaborate, the candidate genes were subjected to three analytical approaches: support vector machines with recursive feature elimination (SVM-RFE) with five-fold cross-validation (implemented using the e1071 package, version 1.7.16) (Zheng et al., 2024), Boruta analysis (conducted via the Boruta package, version 8.0.0) (Liu B. et al., 2025), and eXtreme Gradient Boosting (XGBoost) analysis (carried out using the xgboost package, version 2.1.1.1) (Lu et al., 2025). Ultimately, the overlapping genes from the three machine learning algorithms—defined as key genes—were identified through the ggvenn package (v 0.1.10). Following this, expression profiles were systematically compared between keloid and normal samples in GSE113619 and GSE158395. A comparison of the differential expression of key genes between keloid and normal samples was conducted via the Wilcoxon test, with significance defined as p < 0.05. Key genes that exhibited significant differential expression between the two groups and maintained consistent expression patterns in both GSE113619 and GSE158395 were selected as hub genes, with visualization performed using the ggplot2 package (v 3.5.1).

### 2.5 Filtering of instrumental variables (IVs)

The causal link between the hub genes and keloids was validated through MR analysis. For the MR analysis, eQTLs associated with hub genes were considered exposure factors, and keloids were regarded as the outcome. The TwoSampleMR package (v 0.6.8) (Hemani et al., 2018) was used to carry out the MR analysis. Three assumptions had to be fulfilled by the IVs in the MR analysis: 1) A key component of the relevance assumption is the expectation that SNPs are strongly correlated with exposure factors; 2) For the independence assumption, the key premise is that SNPs are independent of factors that act as confounders; 3) The exclusion restriction assumption—SNPs are assumed to impact the outcome exclusively through exposure factors. Based on the assumptions of MR, the extract_instruments function was used to find SNPs significantly associated with the exposure factor (p < 1 × 10^−6^). Subsequently, we set the clump option to TRUE, which served to remove SNPs exhibiting linkage disequilibrium (Hammad et al., 2020) under the criteria of R^2^ = 0.001 and kb = 100. Afterward, drawing on the outcome GWAS data and the pre-screened IVs, IVs with a significant association to the outcome were eliminated (proxies = TRUE; rsq = 0.8). At least two SNPs associated with the exposure factor were retained. Meanwhile, SNPs with F < 10 were excluded (Burgess and Thompson, 2011). Finally, through the harmonise_data function, effect alleles and effect sizes were standardized, paving the way for further analysis.

### 2.6 MR analysis and sensitivity analysis

Five methods were used for MR analysis in this study through the mr function: MR Egger (Bowden et al., 2015), weighted median (Bowden et al., 2016), inverse variance weighted (IVW) (Burgess et al., 2015), simple model (Hemani et al., 2018), and weighted model (Hartwig et al., 2017). IVW was utilized as the primary reference method for MR analysis (p < 0.05) (Burgess et al., 2013). The forest plot was drawn using the forest function from the forestploter package (version 1.1.2) (Deng et al., 2022). Keloid risk factors were defined as genes with an odds ratio (OR) greater than 1, while protective factors were genes with an OR less than 1 (Chu et al., 2025). The mr_scatter_plot function was then employed to generate a scatter plot, serving to analyze the correlation between exposure factors and the outcome. A forest plot—showing the diagnostic validity of exposure factors for the outcome—was generated using the mr_forest_plot function, and a funnel plot to check if the MR analysis conformed to Mendel’s second law was created via the mr_funnel_plot function. Moreover, to evaluate the reliability of the MR findings, a sensitivity analysis was conducted, which involved running a heterogeneity test (Cochran’s Q test) using the mr_heterogeneity function (with p > 0.05) (Greco et al., 2015; Bowden et al., 2019). It is important to note that the fixed-effects IVW approach applied for p > 0.05, in contrast to the random-effects IVW method used when the p-value did not exceed 0.05. A horizontal pleiotropy test (with p > 0.05) was conducted via the mr_pleiotropy_test function, whereas the mr_leaveoneout function facilitated a leave-one-out (LOO) analysis. The findings from the MR analysis informed the subsequent steiger test, which utilized the steiger_filtering function in the TwoSampleMR package (v 0.6.8) to determine the directionality between exposure factors and the outcome. The conditions for passing the steiger test were that the correct causal direction was TRUE and p < 0.05 (Kong et al., 2024).

### 2.7 Molecular regulatory network of hub genes

To demonstrate how hub genes regulate each other, a molecular regulatory network for them was established. Initially, the microRNA Data Integration Portal (mirDIP) database (version 4.1, accessible at http://ophid.utoronto.ca/mirDIP/) was utilized to predict the microRNAs (miRNAs) that regulate the hub genes. Cytoscape software (v 3.10.3) was used to visualize the miRNA-mRNA interaction pairs (Liu P. et al., 2025). The ENCODE database (Encyclopedia of DNA Elements, accessible at https://www.encodeproject.org/) was then used to predict the transcription factors (TFs) that control the hub genes. The mRNA-TF regulatory network was visualized using Cytoscape software (version 3.10.3). In the end, the National Center for Biotechnology Information (NCBI) database (https://www.ncbi.nlm.nih.gov/) served to search for the promoter regions of the genes. It is generally considered that the region from 2,000 bp upstream of the gene start site to 100 bp downstream is a potential promoter region. Subsequently, the JASPAR CORE (JASPAR) database (https://jaspar.genereg.net/) was employed to predict transcription factor transcription factor binding sites (TFBS). Moreover, visualization of the TFBS-hub gene regulatory network was performed using Cytoscape software (version 3.10.3).

### 2.8 Gene set enrichment analysis (GSEA)

GSEA was first carried out using the samples (keloid and normal) from the GSE113619 dataset. Next, the cor function from the stats package (v 4.3.1) in R software (v 4.2.2) was used to perform Spearman correlation analysis between each hub gene and all other genes, resulting in the acquisition of correlation coefficients. After sorting the correlation coefficients in the order of largest to smallest, the ranked results served as the basis for subsequent analytical steps. Later, GSEAs were implemented with the clusterProfiler package (v 4.8.3), where the reference was c2. cp.all.v2022.1. Hs.symbols.gmt downloaded from the Molecular Signatures Database (MsigDB) (http://www.gsea-msigdb.org/gsea/msigdb/index.jsp). To identify enriched pathways, two threshold criteria were used: first, the absolute normalized enrichment score (|NES|) > 1, and second, the p-value <0.05. Afterward, the top five pathways—ranked in descending order based on |NES|—were displayed using the enrichplot package (v 1.20.3).

### 2.9 Immune infiltration analysis

Analyses of immune infiltration were performed on the keloid and normal samples within GSE113619. The infiltration proportions of 28 immune cell types were assessed using the GSVA package (v 1.53.28) The infiltration abundances of immune cells were visualized using the ggplot2 package (v 3.5.1). Afterwards, the Wilcoxon test helped identify immune cells with significant differences between the two groups (p < 0.05), which were then designated as differential immune cells. To present the differential immune cells, a box plot made using the ggplot2 package (v 3.5.1) was employed. The psych package (v 2.4.6.26) facilitated the performance of the Spearman correlation analysis Notably, two sets of correlation analyses were conducted—between differential immune cells, and between hub genes and differential immune cells—using a minimum threshold (|correlation coefficient| > 0.3 and p < 0.05) to ensure the associations were both robust and meaningful. The ggplot2 package (v 3.5.1) generated a correlation heatmap displaying associations between hub genes and differential immune cells. The correlation heatmap of differential immune cells was produced with the pheatmap package (v 1.0.12).

### 2.10 GeneMANIA analysis and drug prediction

To delve deeper into the interactions and functional associations between hub genes and genes that share similar functions, by importing the hub genes into the GeneMANIA online database (http://genemania.org/) and selecting the suitable species “*Homo sapiens*”, an interaction network diagram was constructed.

In the process of predicting potential therapeutic drugs for keloids, the first step was to use the Drug-Gene Interaction database (DGIdb) (https://dgidb.org/) to pinpoint drugs acting on the hub genes. Afterward, Cytoscape software (v 3.10.3) was used to construct and visualize the interaction network between drugs and hub genes. Moreover, a circular plot for the hub genes—designed to display their chromosomal distribution—was formulated via the RCircos package (v 1.2.2) (Yang et al., 2019).

### 2.11 scRNA-seq analysis

The single-cell transcriptome dataset underwent filtering via the Seurat package (v 5.1.0) (Zhang et al., 2023). To filter the data, the subset function of the Seurat package (v 5.1.0) was utilized. Selection of high-quality cells was based on the stringent criteria listed below: 200 < nFeature_RNA (Number of gene expressions in cells) < 4,000, nCount_RNA (genic count) < 10,000, and percent. mt (Mitochondrial ratio) < 15%. Subsequently, these high-quality cells were kept and reserved for additional follow-up analysis. Normalization of the filtered data was performed via the NormalizeData function. In each cell, the expression value of each gene was first divided by the total expression of all genes in that cell, and then this quotient was multiplied by 10,000. The result was added 1 before undergoing logarithmic transformation to obtain normalized data. Through analysis of variance, the FindVariableFeatures function was employed to screen out the top 3,000 genes with highly variable features, which were then noted as highly variable genes. The LabelPoints function facilitated visualization of the results, with labels applied to the top 10 genes exhibiting the greatest differences in expression levels. Later, dimensionality reduction was achieved through principal component analysis (PCA). Highly variable genes underwent principal component dimensionality reduction analysis via the runPCA function. The p-values of the principal components (PCs) were calculated based on the permutation test algorithm of the Jackstraw function. Variance inflection values across different PC values were computed using the ElbowPlot function, with the results visualized. The PC at the inflection point in the scree plot was selected for subsequent analyses. Next, the cells were clustered using the Louvain algorithm with the FindClusters function to obtain distinct cell clusters (resolution = 0.4).

### 2.12 Cell annotation and screening of key cells

Employing the FindAllMarkers function, marker genes for each cell cluster were identified under parameters that restricted consideration to positively differentially expressed genes, with a minimum 25% expression proportion in the target cell cluster and a minimum log-fold change of 0.25. The cell clusters were automatically annotated using the SingleR package (v 2.2.0) in combination with the Human Primary Cell Atlas (HPCA) database. By comparing the gene expression data of each cell cluster with the reference database, cell types were assigned to each cluster. Based on this, the cell clusters were manually adjusted and renamed, categorizing them into distinct subgroups. Visualization of the cell cluster distribution was performed with a uniform manifold approximation and projection (UMAP) plot. The ggplot2 package (v 3.5.1) generated a bubble plot to display the expression levels of the selected marker genes across the annotated cell clusters. Based on the clustered and annotated cell cluster data, the cell counts of each cluster in every sample were tallied. A bar plot was then created using the ggplot2 package (v 3.5.1) to illustrate the distribution of each cell cluster across different individuals. For a more precise quantification of how cell cluster proportions differ between the two groups, the Wilcoxon test was applied to compare the mean proportions of each cell cluster (p < 0.05), and the results were visualized using the ggplot2 package (v 3.5.1). To conduct functional enrichment analysis on differential cells, the analyze_sc_clusters function of the ReactomeGSA package (version 1.14.0) (Zhang L. et al., 2024) was employed. The top 10 enriched pathways with the largest differences in pathway activity among differential cells were plotted using the plot_gsva_heatmap function from the ReactomeGSA package (v 1.14.0). Subsequently, a UMAP plot was generated using the FeaturePlot function to visualize the distribution of hub genes across differential cells in the two sample groups. Next, the expression data of mRNAs across different cell populations were obtained using the AverageExpression function, enabling the localization analysis of hub genes within differential cells. To evaluate variations in hub gene expression between control and keloid patient cells, a t-test was carried out (p < 0.05). Ultimately, the Wilcoxon test was utilized to examine hub gene expression in differential cells (p < 0.05), and cells displaying significant differential expression of all hub genes between keloid and normal skin samples were identified as key cells.

### 2.13 Cellular communication and pseudo-time analyses

The CellChat package (v1.6.1) (Luo et al., 2023) was employed to carry out cell communication analysis on the various cell types annotated through the aforementioned steps. The objective was to evaluate cell-cell communication through the detection of known ligand-receptor pair expression within individual cell populations and between different cell populations. The number and weight graphs of cell-cell interactions were obtained, and the differences in the interaction strength between cells in the disease and control groups were determined.

Dimensionality reduction and clustering were performed on key cells, followed by cell annotation of different clusters based on literature-validated marker genes. Through a bubble plot, the expression levels of selected marker genes across the annotated cell types were visualized. which was generated using ggplot2 (v 3.5.1). To examine how gene expression dynamically changes over time during cell state transitions in the single-cell dataset, pseudotime analysis was applied to key cells using the Monocle package (v 2.28.0) (Qiu et al., 2017), thereby projecting all cells within a single population onto a trajectory that includes a root and multiple branches. Various pseudotime plots were then generated to depict the expression patterns of key cell subsets, different states, and hub genes. Expression profiles of specific genes at branch points were also displayed to illustrate differential gene expression across distinct developmental trajectories.

### 2.14 Single cell TF regulation and metabolic pathway analysis

The regulatory roles of transcription factors (TFs) in single cells were examined by constructing a regulatory network via the SCENIC website (v 1.3.1) (http://aertslab.org/#scenic), where the analysis focused on quality-controlled cells and genes. First, the GRNBoost2 algorithm was employed to predict co-expression regulatory networks. Subsequently, TF binding motifs were identified using RcisTarget package (v 1.20.0) (Zhang et al., 2021). Finally, the AUCell package (v 1.22.0) (Holland et al., 2020) was used to score the activity of TF target gene sets in each cell. TFs with p < 0.01 and mean Area Under the Curve (AUC) > 0.1 were selected.

For a deeper exploration of the metabolic traits of hub genes in key cell subtypes, the scMetabolism computational workflow (v 0.2.1) (Liu W. et al., 2025), which quantifies single-cell metabolism, was employed to visualize and assess the metabolic diversity of single cells across each cluster. First, based on the results of previous cell annotation and hub gene analysis, key cell subtypes were screened, and their single-cell expression matrices were extracted. The AUCell package (v 1.22.0) was used by scMetabolism to score each cluster, and the activity scores of clusters within each metabolic pathway were ultimately derived from the conventional single-cell matrix file. A metabolic heatmap was plotted accordingly to observe the activity levels of different metabolic pathways across various key cell subtypes. The top 10 metabolic pathways with the highest activity scores were displayed.

### 2.15 RT-qPCR-mediated validation of target hub genes

To verify the consistency between hub gene expression in clinical samples and bioinformatics results, five pairs of normal and keloid tissue samples were collected from the Department of Plastic Surgery, People’s Hospital of Xinjiang Uygur Autonomous Region. Every participant gave written informed consent, signing and completing the consent forms that were required for the study. Having been reviewed by the Clinical Research Ethics Committee of the People’s Hospital of Xinjiang Uygur Autonomous Region, this study was approved, with the Ethics Opinion Number specified as KY2025082915. Results were visualized and p-values computed with Graphpad Prism 10. The Trizol method (supplied by Vazyme Biotech Co.,Ltd., Nanjing, China) was employed to extract total RNA from the 10 samples. The conversion of total RNA to cDNA via reverse transcription was carried out with HP All-in-one qRT Master Mix II RT203-Ver.1, supplied by Yuneng Biotechnology Co., LTD. (Kunming, China). The following RT-qPCR analysis utilized 2xUniversal Blue SYBR Green qPCR Master Mix (Servicebio, Wuhan, China), with H-GAPDH acting as the internal reference for the quantification of hub gene expression. The PCR primer sequences referenced in this study are presented in Table 1. To assess intergroup expression variations between keloid and normal tissues, the 2^−ΔΔCT^ method was applied. To evaluate expression differences between keloid tissues and normal tissues, a t-test was conducted (p < 0.05).

**TABLE 1:** Primer sequences.

Primer	Sequences
PIK3R3 F	GAAGTATTCCTGGGTGCGCT
PIK3R3 R	GTTGAGGCATCTCGGACCAA
ANGPTL5 F	GATGCATTCCGGGGTCTCAA
ANGPTL5 R	ACCAGCCGGTCTTGTTATGG
Internal reference H-GAPDH F	ATGGGCAGCCGTTAGGAAAG
Internal reference H-GAPDH R	AGGAAAAGCATCACCCGGAG

### 2.16 Statistical analysis

R software (v 4.2.2) was used for analyzing the bioinformatics data, with the Wilcoxon test applied to evaluate differences between the two sample groups (p < 0.05).

## 3 Results

### 3.1 Acquisition of 19 candidate genes

**FIGURE 1:**
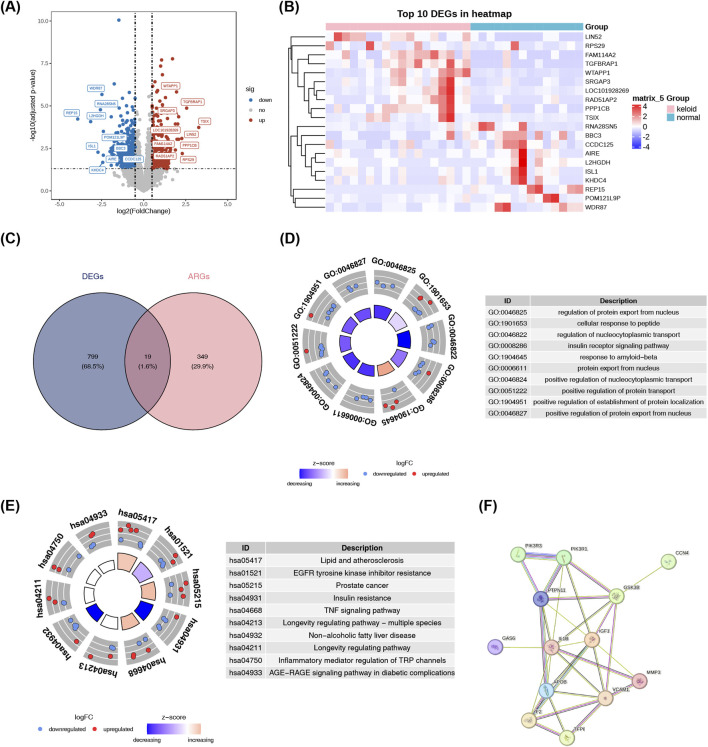
Identification and functional profiling of adipocytokine-related genes in keloid tissues reveals dysregulated signaling networks. **(A)** Genome-wide differential expression analysis reveals extensive transcriptomic alterations in keloids. The volcano plot displays 818 significantly dysregulated genes (311 upregulated, red; 507 downregulated, blue) in keloid versus normal skin tissues from the GSE113619 dataset (18 keloid, 14 control). **(B)** Heatmap of differentially expressed genes. The top color bar annotation: pink represents Keloid samples, blue represents control samples. In the heatmap, the y-axis represents genes, with red indicating highly expressed genes and blue indicating lowly expressed genes. **(C)** Venn diagram of candidate genes. Intersection analysis pinpoints 19 candidate adipocytokine-related genes dysregulated in keloids. **(D)** GO enrichment analysis highlights biological processes, molecular functions, and cellular components associated with the 19 candidate genes, emphasizing their roles in cellular response to peptides and related pathways. **(E)** KEGG pathway analysis identifies significant enrichment in pathways such as “proteoglycans in lipid and atherosclerosis”, linking adipocytokine signaling to keloid pathogenesis. **(F)** PPI network of 13 candidate genes shows 31 interaction pairs, including key interactions such as CCN4-GSK3B. Nodes represent proteins with known or predicted structures; cyan and pink edges denote known interactions, while green, red, and blue edges indicate predicted interactions.

In the GSE113619 dataset (18 patients with keloids, 14 healthy controls), 818 differentially expressed genes were identified through analysis of keloid versus normal samples; among these, 311 were upregulated and 507 were downregulated in keloid samples (Figures 1A,B; Supplementary Table 2). Overlapping these DEGs with ARGs resulted in 19 candidate genes (Figure 1C; Supplementary Table 3). GO enrichment analysis classified these candidate genes into 428 biological processes (BPs), 1 cellular component (CC), and 47 molecular functions (MFs) (Figure 1D; Supplementary Table 4). A notable enrichment of the candidate genes was observed in “cellular response to peptide”. Then, KEGG pathway analysis was conducted, revealing significant enrichment in 102 key enrichment pathways, including proteoglycans in lipid and atherosclerosis (Figure 1E; Supplementary Table 5). Ultimately, a PPI network was built, consisting of the 13 candidate genes (meeting the criterion of an overall score ≥0.4). Those genes were observed to have 31 interactions with each other, including interactions between CCN4 and GSK3B (Figure 1F).

### 3.2 Acquisition of two hub genes

**FIGURE 2:**
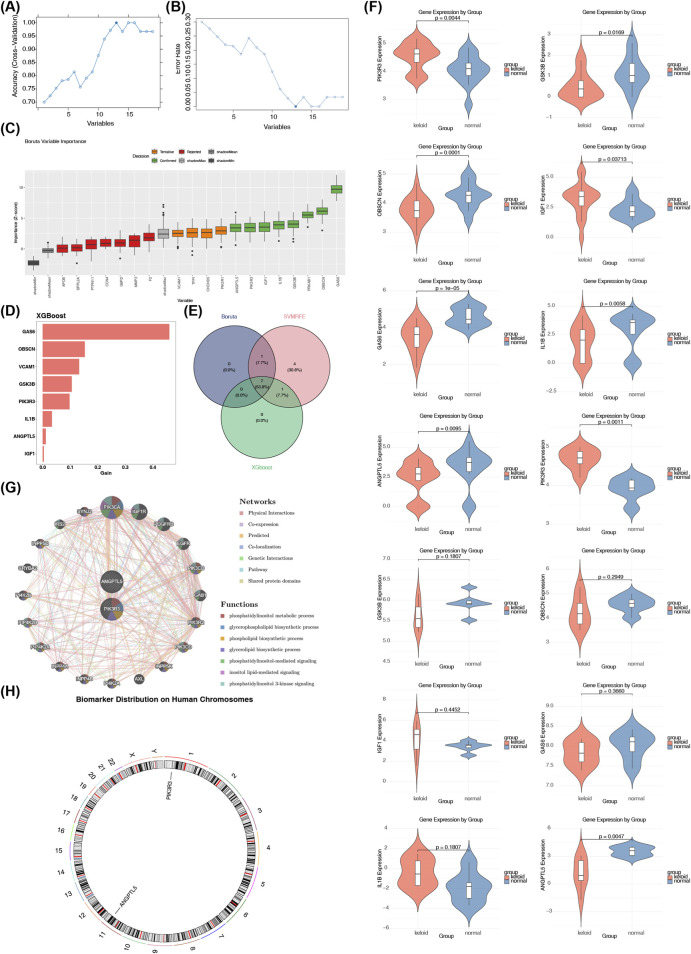
Integrative machine learning identifies PIK3R3 and ANGPTL5 as hub genes for keloids. **(A)** SVM-RFE plot shows classification accuracy (y-axis) as a function of the number of included features (x-axis), with the peak accuracy indicating the optimal feature set. **(B)** Corresponding error rate plot for SVM-RFE highlights the minimal error achieved with the selected feature subset. **(C)** Boruta feature selection analysis identifies eight genes as important predictors, with confirmed (green), tentative (yellow), and rejected (red) features displayed. **(D)** XGBoost analysis ranks gene importance, with the top eight genes contributing most to model performance. **(E)** The intersection of genes selected by SVM-RFE (13 genes), Boruta (8 genes), and XGBoost (8 genes) yielded a consensus set of seven genes: GAS6, IGF1, GSK3B, PIK3R3, IL1B, ANGPTL5, and OBSCN. **(F)** Validation in two cohorts (GSE113619 and GSE158395) confirmed that PIK3R3 is significantly upregulated and ANGPTL5 is significantly downregulated in keloids compared to normal skin (p < 0.05), establishing them as the final hub genes. **(G)** GeneMANIA functional association network illustrates interactions between the two hub genes and 20 related genes (e.g., PIK3CA, IGF1R, PDGFRB), enriched in processes such as phosphatidylinositol metabolic signaling. **(H)** Chromosomal localization map shows PIK3R3 on chromosome 1 and ANGPTL5 on chromosome 11, providing genomic context for further investigation.

In GSE113619, SVM-RFE analysis was carried out, and 13 SVMRFE genes were identified (Figures 2A,B; Supplementary Table 6). Subsequently, Boruta analysis was carried out, and eight Boruta genes were identified (Figure 2C; Supplementary Table 7). Then, XGBoost analysis was carried out, and eight XGBoost genes were identified (Figure 2D; Supplementary Table 8). Following this, an intersection analysis of the SVMRFE genes, Boruta genes, and XGBoost genes was conducted, yielding seven key genes (GAS6, IGF1, GSK3B, PIK3R3, IL1B, ANGPTL5, and OBSCN) (Figure 2E; Supplementary Table 9). We carried out verification of expression levels, and this process covered both GSE113619 and GSE158395 (4 patients with keloids, 6 healthy controls), it was found that PIK3R3 was significantly upregulated in the keloid group, ANGPTL5 was significantly downregulated in the keloid group, and this situation existed in both datasets (p < 0.05). These two genes were found to exhibit consistent and significant expression differences. Therefore, these two genes were regarded as hub genes (Figure 2F). These two hub genes were associated with 20 genes including PIK3CA, IGF1R, and PDGFRB, and their related functions included phosphatidylinositol metabolic process (Figure 2G). PIK3R3 and ANGPTL5 were located on chromosomes 1 and 11, respectively (Figure 2H). Collectively, the integration of three machine learning algorithms identified PIK3R3 and ANGPTL5 as robust hub genes for keloids, with their chromosomal localization, associated genes, and functional roles providing a comprehensive molecular framework for understanding keloid pathogenesis.

### 3.3 PIK3R3 was identified as a risk factor for keloids

**FIGURE 3:**
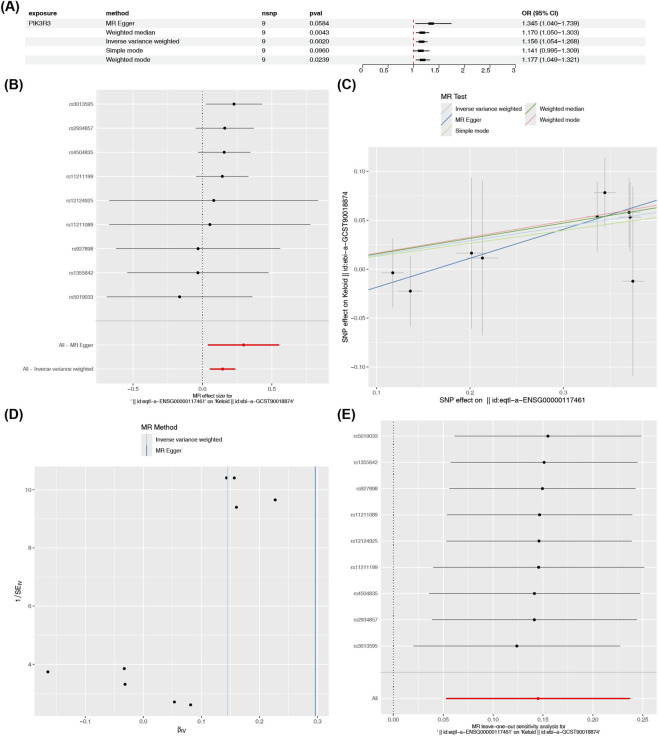
Mendelian randomization identifies PIK3R3 as a causal risk factor for keloids. **(A)** Forest plot compares OR and 95% CI from five Mendelian randomization methods, showing consistent positive association of PIK3R3 with keloid risk using the IVW method as primary reference (OR = 1.156, 95% CI = 1.054–1.268, p = 0.0020). **(B)** Forest plot displays individual IV effect sizes for PIK3R3. **(C)** Scatter plot of SNP-exposure vs. SNP-outcome associations demonstrates positive slope for PIK3R3. **(D)** Funnel plot assesses symmetry of IV estimates; even distribution of SNPs around the IVW line indicates absence of directional pleiotropy and validates MR model assumptions. **(E)** Leave-one-out sensitivity analysis forest plot confirms that no single SNP disproportionately drives the causal estimate, reinforcing the robustness of the PIK3R3–keloid association.

Among the two hub genes, analysis of the publicly available GWAS (IEU OpenGWAS, ID: ebi-a-GCST90018874, comprising 668 cases and 481,244 controls) and eQTL data indicated that PIK3R3 passed the IVW method (p = 0.002) (Figure 3A). Then, PIK3R3 was obtained after being screened through the horizontal pleiotropy test (p = 0.257) (Table 2). Then, PIK3R3 was validated through the Steiger directionality test (causal direction = TRUE and p = 3.26 × 10^−133^) (Table 3). Finally, PIK3R3 associated with keloids was identified. For detailed illustration, the MR results of PIK3R3 (OR = 1.156, 95% confidence interval (CI) = 1.054–1.268, p = 0.0020) were as follows (Figure 3A). We identified PIK3R3 as a risk factor, with the supporting evidence for this conclusion available in the result Figureures. The results of the forest plot indicated that, in the IVW method, the MR effect size of PIK3R3 was greater than zero (Figure 3B). A positive slope was observed for PIK3R3 in the scatter plot, as shown in Figure 3C. Results from the randomness test indicated that the IV of PIK3R3 showed an even distribution on both sides of the IVW line, signifying that the MR analysis conformed to the second law of MR classification (Figure 3D). These results indicated that PIK3R3 had risk associations with keloids. Furthermore, the heterogeneity analysis indicated that all four genes had p-values >0.05, with PIK3R3 showing no evidence of heterogeneity (p = 0.940) as presented in Table 4. Moreover, no confounding factors were found through the horizontal pleiotropy test in the MR results (p = 0.257) (Table 2). No points exhibiting severe bias were identified in the LOO analysis, providing additional support for the robustness of the MR findings (Figure 3E). Finally, the steiger test revealed that the causal relationship in this MR result was correct (causal direction = TRUE and p = 3.26 × 10^−133^) (Table 3). In conclusion, PIK3R3 was identified as a risk factor.

**TABLE 2:** Horizontal pleiotropy test.

id.exposure	id.outcome	Outcome	Exposure	egger_intercept	se	pval
eqtl-a-ENSG00000117461	ebi-a-GCST90018874	Keloid || id:ebi-a-GCST90018874	|| id:eqtl-a-ENSG00000117461	−0.048090214	0.0389321198024114	0.256590884315242

**TABLE 3:** Steiger directional tests.

Gene	Outcome name	Steiger test correct direction	Steiger test pval	SNP	Steiger filtering correct direction	Steiger filtering pval
PIK3R3	keloid	TRUE	3.26e-133	rs11211089	TRUE	2.34e-35
				rs11211199	TRUE	7.04e-206
				rs12124925	TRUE	3.57e-32
				rs1355642	TRUE	3.57e-215
				rs2934857	TRUE	3.37e-168
				rs3013595	TRUE	2.81e-174
				rs4504835	TRUE	3.59e-204
				rs5019033	TRUE	1.61e-23
				rs927898	TRUE	5.37e-21

**TABLE 4:** Heterogeneity test.

id.exposure	id.outcome	Outcome	Exposure	Method	Q	Q_df	Q_pval
eqtl-a-ENSG00000117461	ebi-a-GCST90018874	Keloid || id:ebi-a-GCST90018874	|| id:eqtl-a-ENSG00000117461	MR Egger	1.37611669330461	7	0.986291581353163
eqtl-a-ENSG00000117461	ebi-a-GCST90018874	Keloid || id:ebi-a-GCST90018874	|| id:eqtl-a-ENSG00000117461	Inverse variance weighted	2.90191565443272	8	0.940361143777429

### 3.4 The network of hub genes

**FIGURE 4:**
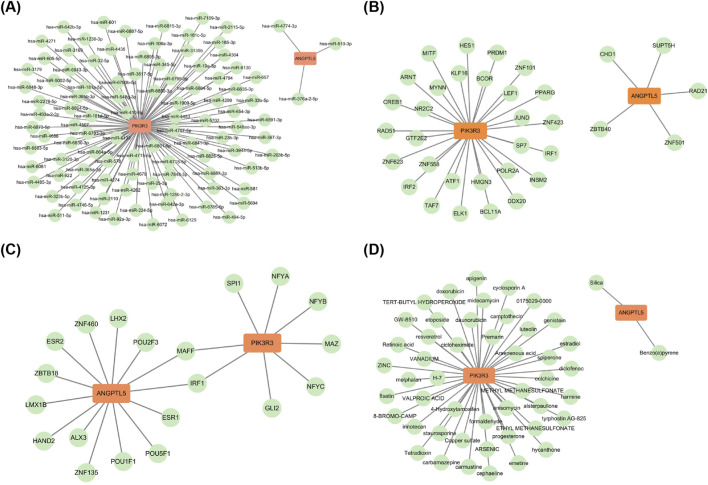
Multi-layer regulatory networks and potential therapeutic targets for PIK3R3 and ANGPTL5 in keloids. **(A)** mRNA-miRNA interaction network shows 97 miRNAs targeting PIK3R3 and 3 targeting ANGPTL5, including key pairs PIK3R3-hsa-miR-363-3p and ANGPTL5-hsa-miR-376a-2-5p (green: miRNAs; orange: hub genes). **(B)** mRNA-TF regulatory network predicts 30 TFs regulating PIK3R3 and 5 regulating ANGPTL5, such as PIK3R3-ZNF263 and ANGPTL5-CHD1 (green: TFs; orange: hub genes). **(C)** TFBS analysis identifies 8 binding sites in PIK3R3 and 14 in ANGPTL5 promoters; MAFF and IRF1 are common to both, suggesting shared transcriptional regulation (green: TFBS; orange: hub genes). **(D)** Drug-gene network reveals 47 candidate drugs for PIK3R3 (e.g., apigenin) and 2 for ANGPTL5 (e.g., silica), highlighting potential therapeutic options (green: drugs; orange: hub genes).

A total of 97 miRNAs targeting PIK3R3 and three miRNAs targeting ANGPTL5 were predicted using bioinformatics databases. The mRNA-miRNA regulatory network consisted of 102 nodes and 100 interactions, including PIK3R3-hsa-miR-363-3p and ANGPTL5-hsa-miR-376a-2-5p (Figure 4A; Supplementary Table 10). Subsequently, 30 TFs targeting PIK3R3 and 5 TFs targeting ANGPTL5 were predicted. The mRNA-TF regulatory network was composed of 37 nodes and 35 interaction relationships, including PIK3R3-ZNF263 and ANGPTL5-CHD1 (Figure 4B; Supplementary Table 11). Then, eight TFBSs in the promoter region of PIK3R3 and 14 TFBSs in the promoter region of ANGPTL5 were screened. MAFF and IRF1 were identified as the common TFBS for the two hub genes. The TFBS-hub gene regulatory network consisted of 24 nodes and 22 interacting edges, including PIK3R3-MAFF and ANGPTL5-MAFF (Figure 4C; Supplementary Table 12). Finally, 47 potential therapeutic drugs, such as apigenin, were predicted for PIK3R3 from drug databases, while two drugs, such as silica, were identified for ANGPTL5 (Figure 4D; Supplementary Table 13). Collectively, these integrated analyses of miRNAs, TFs, TFBSs, and drug targets comprehensively delineated the regulatory networks and therapeutic potential of PIK3R3 and ANGPTL5 in keloids, providing a multi-faceted molecular foundation for mechanistic studies and drug development. It is important to note that these drug predictions are preliminary and should be regarded as hypothesis-generating, identifying potential candidates for future mechanistic and preclinical validation rather than as established therapeutic options.

### 3.5 Strong correlations were observed among differential immune cells

**FIGURE 5:**
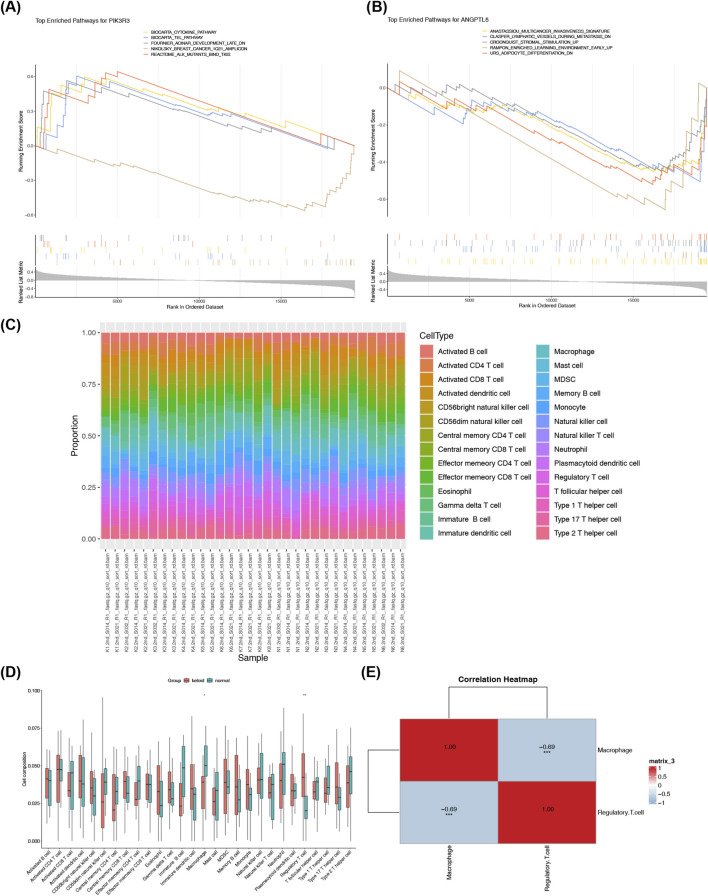
GSEA and immune infiltration analyses identify keloid-associated pathways and altered immune cell profiles. **(A)** GSEA for PIK3R3 reveals enrichment in 261 pathways including the Biocarta Cytokine Pathway, implicating inflammatory signaling in keloids. **(B)** GSEA for ANGPTL5 shows 337 enriched pathways such as the URS Adipocyte Differentiation DN set, linking it to adipocyte-related processes. **(C)** Immune infiltration profiling in keloid (n = 18) vs. normal (n = 14) samples highlights activated dendritic cells as highly abundant in both groups. **(D)** Macrophages and regulatory T cells differ significantly between keloid and normal tissues (p < 0.05), indicating their role in keloid immune dysregulation. **(E)** Correlation analysis identifies a strong negative association between macrophages and regulatory T cells (cor = −0.69, p = 2.44 × 10^−5^), suggesting immune network coordination in keloids.

In the GSEA analysis (GSE113619), 261 pathways, including biocarta cytokine pathway, were predicted for PIK3R3, while 337 pathways, such as URS adipocyte differentiation DN, were identified for ANGPTL5 (Figures 5A,B; Supplementary Tables 14, 15). As shown in Figure 5C, activated dendritic cells exhibited a relatively high abundance of infiltration in both keloid and normal skin samples (GSE113619). Significant differences were observed in two specific immune cell populations including macrophages and regulatory.T.cells (p < 0.05) (Figure 5D). Correlation analysis indicated that there were significant correlations between macrophages and regulatory.T.cells (cor = −0.69, p = 2.44 × 10^−5^) (Figure 5E; Supplementary Table 16). Taken together, these findings identified the distinct pathway enrichments of PIK3R3 and ANGPTL5, along with key characteristics and correlations within specific immune cell populations. Such findings contribute to enhancing the overall understanding of the immune processes and molecular mechanisms underlying keloid development.

### 3.6 Identification of seven cell types

**FIGURE 6:**
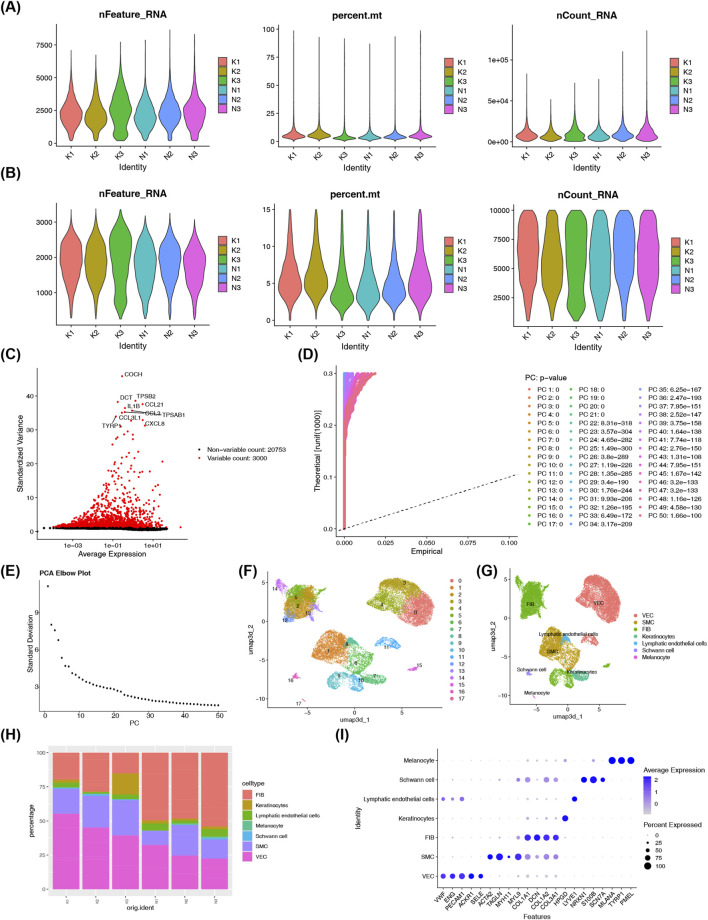
Single-cell RNA sequencing identifies seven major cell populations in keloid and normal scar tissues. **(A)** Pre-quality control metrics (nFeature, nCount, percent. mt) across samples from GSE163973 (3 keloid, 3 normal scar samples). **(B)** Post-quality control metrics after filtering cells with 200 < nFeature <4,000, nCount <10000, and percent. mt < 15%. **(C)** Highly variable gene plot: red dots denote the top 3,000 variable genes used for downstream analysis. **(D)** PCA plot shows separation of cell populations based on transcriptional variation. **(E)** Scree plot indicates the first 40 principal components capture the majority of biological variation. The x-axis represents the number of principal components, and the y-axis represents the standard deviation of principal component scores. **(F)** Unsupervised clustering identifies 18 initial cell clusters (resolution = 0.4). **(G)** UMAP visualization of seven annotated cell types: vascular endothelial cells, smooth muscle cells, fibroblasts, keratinocytes, lymphatic endothelial cells, Schwann cells, and melanocytes. **(H)** Cell type abundance varies between keloid and normal individuals, with VEC enriched in keloid and FIB enriched in normal tissue. **(I)** Marker gene expression bubble plot confirms cell type identity, with color intensity indicating average expression and dot size representing expression percentage.

In the single-cell transcriptomic dataset GSE163973 from the GEO database (comprising three keloid scar tissue samples and three normal scar tissue samples), a total of 48,524 cells and 23,753 genes were detected prior to filtering. After strict cell screening, the final dataset included 27,432 cells and 23,753 genes (Figures 6A,B). The top 3,000 highly variable genes and Top10 genes were identified and used for subsequent analysis (Figure 6C). Following PCA, it was observed that the standard deviation stabilized significantly beyond the first 40 PCs, suggesting that the majority of the true biological signal was captured within these initial components. In turn, 40 PCs (the first set) were picked for downstream analytical processes (Figures 6D,E). Through clustering analysis, a total of 18 distinct cell clusters were identified, each representing a unique cell population based on their transcriptional profiles (resolution = 0.4) (Figure 6F). These clusters were subsequently annotated to known cell types as vascular endothelial cells (VEC), smooth muscle cells (SMC), fibroblast cells (FIB), keratinocytes, lymphatic endothelial cells, schwann cells, and melanocytes using acquired marker genes (Figure 6G; Supplementary Table 17). VECs were found to be more distributed in individuals with keloids, while FIBs were more abundant in individuals with normal skin tissues (Figure 6H). Simultaneously, the marker genes for each separate cell type were found to display a comparatively high level of specific expression (Figure 6I). Overall, the single-cell transcriptome analysis successfully filtered and processed the data, identified highly variable genes and principal components, clustered and annotated distinct cell populations, and revealed differences in cell distribution between keloid and normal tissues, laying a foundation for exploring the cellular heterogeneity and specific biological characteristics underlying keloid formation.

### 3.7 Identification of key cells

**FIGURE 7:**
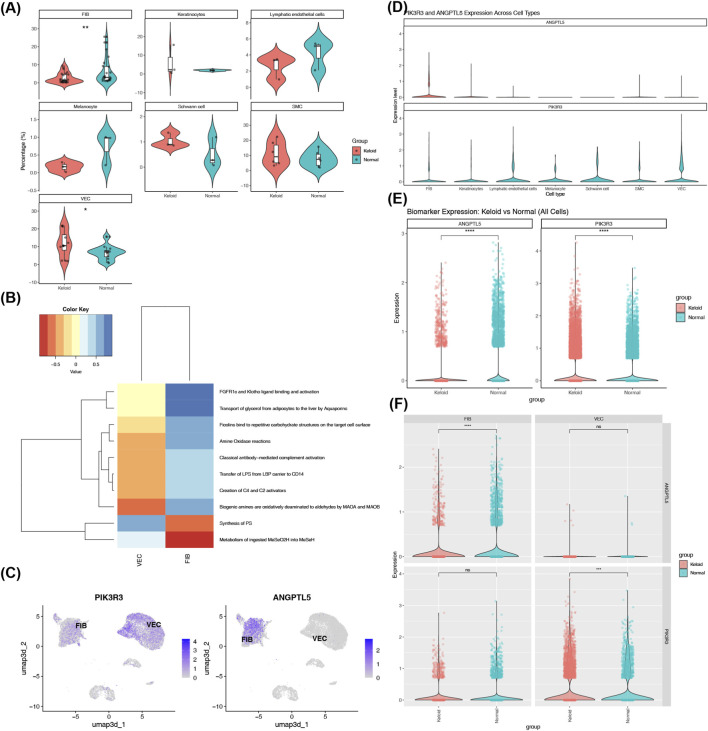
Fibroblasts are identified as key cells expressing hub genes in keloid pathogenesis. **(A)** Comparison of cell cluster proportions between keloid (red) and normal (blue) samples identifies fibroblasts and vascular endothelial cells as differentially abundant cell populations (p < 0.05). **(B)** Functional enrichment analysis of differentially abundant cells reveals 1,725 enriched pathways, including amine oxidase reactions. **(C)** UMAP plots show PIK3R3 distributed in both FIB and VEC, while ANGPTL5 expression is primarily confined to FIB (color intensity reflects expression level). **(D)** Expression profiles of ANGPTL5 (upper) and PIK3R3 (lower) across annotated cell clusters confirm high expression of both hub genes in fibroblasts. **(E)** Aggregate expression analysis indicates PIK3R3 is significantly upregulated and ANGPTL5 is downregulated in keloid compared to normal cells across all cell types (p < 0.05). **(F)** Subtype-specific analysis confirms ANGPTL5 expression is significantly altered in FIB, while PIK3R3 expression differs significantly in VEC between keloid and normal tissues.

It was found that the 2 cell clusters, FIB and VEC, showed differences between keloid patients and healthy controls (p_FIB_ < 0.01, p_VEC_ < 0.05) (Figure 7A). The functional enrichment analysis on differential cells revealed 1,725 enriched pathways, with amine oxidase reactions being one of them (Figure 7B; Supplementary Table 18). PIK3R3 was found to be distributed in both FIBs and VECs, while ANGPTL5 was only distributed in FIBs (Figure 7C). Additionally, we found that PIK3R3 was expressed across all cell clusters, while ANGPTL5 showed high expression exclusively in FIBs (Figure 7D). When analyzing all cells, PIK3R3 and ANGPTL5 showed significant expression differences between the control group and keloid patients. PIK3R3 expression was significantly higher in the keloid group relative to the control group, whereas the opposite was true for ANGPTL5—its expression was notably higher in the control group than in the keloid group (Figure 7E). For the FIB cluster, keloid skin samples and normal skin samples differed significantly in ANGPTL5 expression; in the VEC cluster, by contrast, significant differences between the two sample types were detected in PIK3R3 expression (Figure 7F). In terms of overall expression, both hub genes were highly expressed in FIBs. Therefore, FIBs were defined as the key cells.

### 3.8 Cellular communication and cellular differentiation trajectory

**FIGURE 8:**
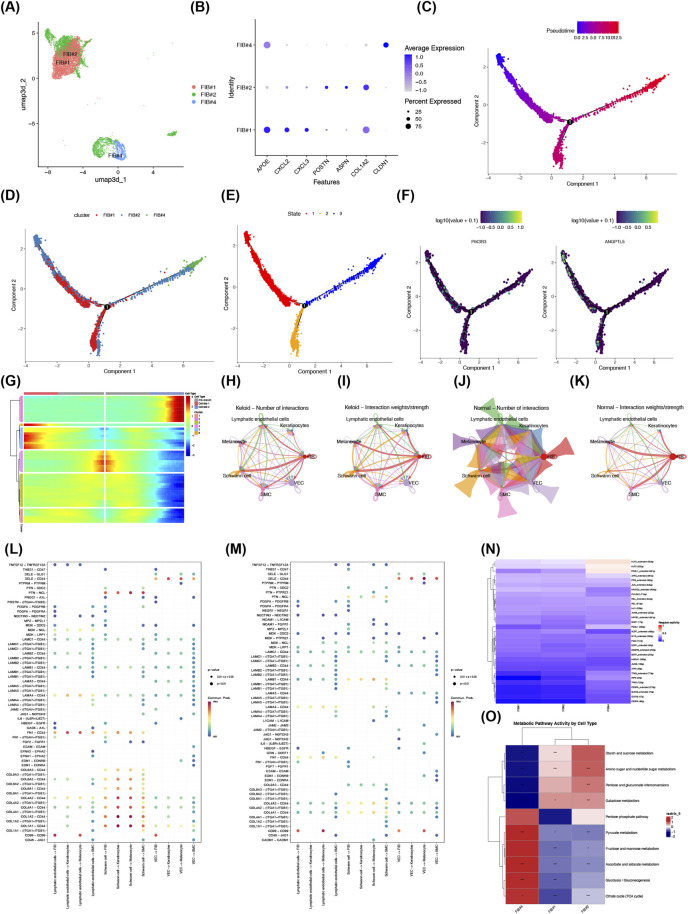
Single-cell analysis reveals fibroblast heterogeneity, differentiation trajectories, intercellular communication, and metabolic activity in keloids. **(A)** UMAP plot of fibroblasts subclustered into three subtypes (FIB#1, FIB#2, FIB#4) from keloid and normal samples. **(B)** Marker expression bubble plot confirms subtype identity: FIB#1 expresses pro-inflammatory genes, FIB#2 mesenchymal markers, and FIB#4 secretory papillary fibroblast markers. **(C)** Pseudotime trajectory orders fibroblasts from early (blue) to late (red) differentiation states. **(D)** Distribution of fibroblast subtypes along pseudotime shows FIB#1 and FIB#2 predominant early, and FIB#4 enriched late. **(E)** Differentiation state map highlights three distinct developmental stages. **(F)** Dynamic expression of PIK3R3 and ANGPTL5 declines over pseudotime, with ANGPTL5 higher in early subtypes. **(G)** Branch-point analysis identifies gene clusters associated with distinct differentiation fates. **(H–K)** Cell-cell interaction networks show strong fibroblast-Schwann cell communication in keloids, and fibroblast-melanocyte interaction in normal tissue. Bubble plots highlight CD99^−^CD99 as a key ligand-receptor pair reduced in normal tissue. **(L,M)** Ligand‑receptor pair bubble plots illustrating cell‑cell communication in keloid and normal tissues. Color represents communication probability, and bubble size corresponds to statistical significance (p < 2.0 × 10^-20^, p < 2.0 × 10^-100^, p < 1 × 10^-50^). **(N)** Transcription factor activity heatmap identifies 31 TFs differentially active across fibroblast subtypes (p < 0.01, AUC >0.1). **(O)** Metabolic pathway activity across fibroblast subtypes shows enrichment in starch/sucrose, galactose, and pentose phosphate pathways. The x-axis shows fibroblast subtypes, and the y-axis shows the top ten metabolic pathways by score. Red indicates positive and blue indicates negative correlations.

We performed secondary dimensionality reduction and clustering on FIBs, with this process resulting in the classification of three subtypes (Figure 8A). High expression of pro-inflammatory cytokine genes was observed in FIB#1 cells, while three known markers were highly expressed in the mesenchyme of FIB#2, and FIB#4 was identified as secretory papillary fibroblasts (Figure 8B; Supplementary Table 19). All cells were then projected onto a root with two branches, and the cells were observed to enter a differentiated state sequentially from left to right (Figure 8C). In the early stage, FIBs mainly existed in the forms of FIB#1 and FIB#2, while in the late stage, an additional form, predominantly derived from FIB#4, was observed (Figure 8D). A total of three different differentiation states were identified in the cells, with each state marked by a distinct color, and the red color represented the earliest differentiation type (Figure 8E). The PIK3R3 gene was found to be expressed throughout the differentiation process of FIBs, whereas ANGPTL5 was more highly expressed in the pre-differentiated FIB#1 and FIB#2 cell subtypes; both genes showed a tendency of decreased expression over time (Figure 8F). At branch point 1, specific genes were divided into 6 distinct subclusters and 3 cell types. Only cluster 4 was highly expressed in pre-branch cells, cluster 5 was highly expressed in cell fate 1, and cluster 2 was highly expressed in cell fate 2 (Figure 8G). In the disease group, FIBs and Schwann cells had the highest number and weight of interactions. On the other hand, the control group showed that FIBs and melanocytes had the strongest interactions in terms of both number and weight (Figures 8H–K). The primary receptor-ligand pair involved in the interaction from Lymphatic endothelial cells to FIBs was identified as CD99 - CD99, and the role of this pair was diminished in the control group (Figures 8L,M). In the single-cell transcription factor regulation analysis, 31 TFs were identified based on p < 0.01 and mean AUC >0.1, most of which exhibited low regulatory activity across the three FIB cell subgroups (Figure 8N; Supplementary Table 20). The metabolic pathways with the highest activity included starch and sucrose metabolism, galactose metabolism, and the pentose phosphate pathway (Figure 8O; Supplementary Table 21). Collectively, these findings comprehensively revealed the subtype characteristics, differentiation dynamics, intercellular communication, transcription factor regulation, and metabolic pathway activity of FIBs, constructing a multi-level cellular regulatory framework for understanding the role of FIBs in keloids progression.

### 3.9 Concordant expression of hub genes in bioinformatics analysis and clinical samples

**FIGURE 9:**
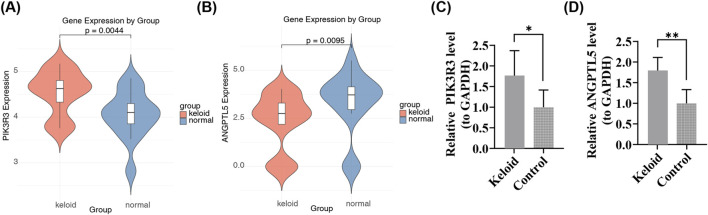
Experimental validation confirms PIK3R3 upregulation in keloids but reveals discordant ANGPTL5 expression in clinical samples. **(A)** Transcriptomic analysis shows PIK3R3 is significantly upregulated in keloid tissues compared to normal skin in the training dataset (GSE113619, p < 0.05). **(B)** Transcriptomic analysis shows ANGPTL5 is significantly downregulated in keloid tissues compared to normal skin in the training dataset (GSE113619, p < 0.05). **(C)** RT-qPCR validation in five paired clinical samples confirms PIK3R3 upregulation in keloid tissues (p < 0.05), consistent with bioinformatic predictions. **(D)** RT-qPCR analysis shows ANGPTL5 is upregulated in clinical keloid samples (p < 0.05), contrary to transcriptomic predictions, potentially due to limited sample size or microenvironmental variability.

In the expression level validation (GSE113619), PIK3R3 was found to be upregulated in keloids, while ANGPTL5 was downregulated in keloids (Figures 9A,B). However, in clinical RT-qPCR, both PIK3R3 and ANGPTL5 were observed to be upregulated in keloids (Figures 9C,D). PIK3R3 showed consistency between bioinformatics predictions and clinical sample validation, whereas the inconsistent expression of ANGPTL5 might have been caused by the small number of clinical samples.

## 4 Discussion

Keloid is a pathological fibroproliferative disorder resulting from abnormal wound healing, characterized by excessive fibroblast proliferation and collagen deposition (Gong et al., 2025). Adipocytes secrete a class of bioactive molecules known as adipocytokines (e.g., leptin, adiponectin), which are critical for regulating metabolism and mediating inflammatory pathways. The relationship between adipocytokines and keloid formation centers on the anti-fibrotic effects of certain adipocytokines. For instance, adiponectin can reduce collagen deposition and scar formation by suppressing fibroblast activation, downregulating pro-fibrotic factors like TGF-β1, and enhancing the expression of anti-fibrotic mediators. Moreover, their ability to modulate inflammatory responses further aids in inhibiting scar progression. Thus, specific adipocytokines represent potential regulatory factors in scar pathogenesis (Darmawan et al., 2020).

By integrating differential expression analysis, machine learning techniques, and experimental validation, we successfully identified PIK3R3 and ANGPTL5 as two key hub genes in this study. MR was used to examine the causal relationship between these hub genes and keloid. Further follow-up analyses, encompassing GSEA, immune infiltration evaluation, and drug prediction, were carried out to uncover the potential mechanisms underlying adipocytokines’ role in keloid development. Using publicly available single-cell RNA sequencing data (GSE163973), we assessed the expression patterns of hub genes across key cell types, providing deeper insight into the molecular landscape of keloids. Finally, RT-qPCR was performed to validate the consistency between bioinformatic predictions and gene expression levels in clinical samples.

Notably, a discordance was observed: while transcriptomic analysis indicated a downregulation of ANGPTL5 in keloid tissues, RT-qPCR results revealed an upregulation. This discrepancy may be attributed to sample heterogeneity (differences in cellular composition between public datasets and clinical samples), post-transcriptional regulation, technical variations (sensitivity and normalization differences between sequencing and RT-qPCR), and biological complexity (ANGPTL5 expression may be dynamically regulated by the microenvironment and disease stage). Future studies with expanded sample sizes are required to definitively elucidate its expression pattern. Serving as a key regulatory subunit of the PI3K/AKT signaling pathway, PIK3R3 is tightly implicated in controlling core cellular processes including proliferation, survival and invasion. Aberrant activation of this pathway has been well documented in various cancers (Suzuki et al., 2025). Notably, the PI3K/AKT pathway also plays a central role in pathological fibrosis by promoting fibroblast activation and collagen synthesis (Liu J. et al., 2025). It is critical to note that keloids are benign fibroproliferative disorders with no neoplastic potential, and the link between PIK3R3 and cancer-related processes does not imply tumorigenesis in keloids. Although PIK3R3 has not been previously linked to keloids, our study demonstrates its significant upregulation in keloid tissues. Mendelian randomization and pathway enrichment analyses further identified PIK3R3 as a potential risk factor associated with cell proliferation-related processes, and this elevated expression was validated in clinical specimens via RT-qPCR. We therefore hypothesize that the high expression of PIK3R3 in keloids may excessively activate the PI3K/AKT signaling pathway, mimicking its pro-growth effects in tumors in a benign cellular context to induce abnormal fibroblast proliferation and ECM deposition.

Our single-cell transcriptomic analysis provides cellular-level evidence for the role of PIK3R3 in keloids. The results show that although PIK3R3 is expressed across various cell types, its expression exhibits the most significant disease-related correlation with fibroblasts and endothelial cells. In fibroblasts, PIK3R3 expression is closely linked to cell activation and differentiation states, supporting the hypothesis that it drives fibrosis by directly regulating fibroblast functions, such as proliferation and ECM synthesis. Notably, PIK3R3 is also significantly upregulated in endothelial cells. Given the well-established connection between pathological fibrosis and angiogenesis (Li et al., 2023), PIK3R3 may indirectly remodel the tissue microenvironment by affecting endothelial cell function, thereby collaboratively promoting keloid progression. In summary, we propose that PIK3R3 primarily exerts its effects directly on fibroblasts, driving their fibrotic phenotype. At the same time, other dermal cell types, such as endothelial cells, may serve as key cooperative targets, collectively forming a pro-fibrotic multicellular regulatory network.

In this study, ANGPTL5 expression was found to be downregulated in keloid tissues. This gene is highly expressed in adipose tissue and is involved in metabolic and immune regulation (Hammad et al., 2020). Notably, changes in ANGPTL5 expression have also been associated with the progression of various cancers, including breast cancer (Yang et al., 2021; Lee et al., 2025). This association reflects a common molecular parallel between benign fibroproliferative diseases and malignancies, which often share overlapping signaling programs related to growth and immune regulation without implying malignant transformation in keloids. Additionally, the GSEA results from this study suggest that ANGPTL5 is linked to adipocyte differentiation pathways. We hypothesize that the downregulation of ANGPTL5 may disrupt the local paracrine homeostasis mediated by adipose tissue. Given the crucial role of adipocytokines in regulating inflammation and fibrosis (Saxena and Anania, 2015), this downregulation could impair potential antifibrotic or anti-inflammatory functions, thereby indirectly shaping a microenvironment that promotes keloid fibrosis. This mechanism mirrors the stromal cell dysfunction observed in certain tumor microenvironments, which similarly promotes disease progression via conserved molecular regulatory mechanisms in a non-malignant setting.

Results from this study indicate that there are notable differences in the infiltration of three immune cell types (immature B cells, macrophages, and regulatory T cells) when comparing keloid samples to normal skin samples. Among these, macrophages and regulatory T cells showed the highest correlation. Serving as critical parts of the innate immune system, macrophages contribute to both the regulation of inflammation and the execution of central roles in tissue repair and fibrotic processes (Zhang J. et al., 2024). It is noteworthy that macrophages exhibit considerable plasticity, and their phenotypic switching directly influences the balance between tissue regeneration and scar formation. Earlier studies have demonstrated that macrophages contribute to the transdifferentiation of fibroblasts into myofibroblasts by way of the TGF-β/Smad3 signaling pathway, thereby intensifying skin fibrosis (Jia et al., 2025). Thus, the polarization states of macrophages are considered crucial in the fibrotic progression of keloid disorders (Dirand et al., 2024). Given these mechanisms, macrophage dysfunction likely contributes substantially to the initiation and development of keloids. Further elucidation of their regulatory networks and cellular interactions may provide novel molecular targets and intervention strategies for clinical therapy.

This study identifies fibroblasts as playing a central role in the pathological microenvironment of keloids. As a typical cutaneous fibroproliferative disorder, keloids are primarily characterized by excessive deposition of pathological ECM and aberrant activation and proliferation of fibroblasts within the dermal tissue (Jin et al., 2025; Zhan et al., 2025). At the molecular level, circular RNA CircGLIS3 exhibits transient upregulation during wound healing, while it exhibits sustained upregulation in keloid tissues, suggesting its potential role in regulating fibroblast function (Niu et al., 2025). Furthermore, studies indicate that melanin accumulation may disrupt normal communication among keratinocytes, melanocytes, and fibroblasts by inducing intracellular iron overload and inhibiting ferroptosis, ultimately amplifying fibrotic phenotypes (Shi et al., 2025). Collectively, this evidence underscores the importance of fibroblasts not only structurally but also in signaling processes that drive keloid progression. Further elucidation of their molecular regulatory networks and cellular interactions may facilitate the development of novel therapeutic strategies targeting these cells, thereby offering new avenues for clinical intervention.

In conclusion, this multi-step integrative analysis identified PIK3R3 and ANGPTL5 as potential hub genes closely associated with keloid pathogenesis. The bioinformatics findings suggest that dysregulated adipocytokine-related pathways and alterations in the immune microenvironment may contribute to disease progression, thereby supporting the roles of PIK3R3 and ANGPTL5 as potential therapeutic targets involved in keloid-associated signaling pathways. These findings deepen our understanding of the pathological processes underlying keloid formation and provide a theoretical basis for the development of targeted therapeutic strategies.

Nevertheless, several limitations should be acknowledged. First, the present analyses were primarily based on transcriptomic data and lacked validation at the protein or metabolic levels. In addition, functional experiments were not conducted to elucidate the precise mechanisms by which these hub genes regulate keloid development. Future studies should therefore incorporate both *in vitro* and *in vivo* functional assays to clarify the regulatory roles of PIK3R3 and ANGPTL5 in fibroblasts and to advance their translational potential as therapeutic targets. Second, owing to resource constraints, experimental validation in this study was limited to two key genes, PIK3R3 and ANGPTL5. Subsequent investigations may be extended to additional candidate genes to systematically delineate their functional networks in keloid pathogenesis. Finally, the Mendelian randomization data used in this study were derived from GWAS conducted in European populations, whereas the transcriptomic and clinical samples were obtained from a Chinese population. Differences in genetic background across populations may influence the generalizability of the findings. Future studies involving multi-population cohorts are therefore warranted to further validate these results and to improve their reliability and potential clinical applicability.

## Data Availability

Publicly available datasets were analyzed in this study. This data can be found here: The datasets (GSE113619, GSE158395, GSE163973) analysed during the current study are available in the Gene Expression Omnibus (GEO) repository, (https://www.ncbi.nlm.nih.gov/gds). The genetic variation data for keloids (identifier: ebi-a-GCST90018874) from the Integrative Epidemiology Unit Open genome-wide association studies (IEU OpenGWAS) website at https://gwas.mrcieu.ac.uk/.

## Glossary

ARG: Adipocytokine-Related Gene
MR: Mendelian Randomization
GSEA: Gene Set Enrichment Analysis
scRNA-seq: Single-Cell RNA Sequencing
RT-qPCR: Reverse Transcription Quantitative Polymerase Chain Reaction
PIK3R3: Phosphoinositide-3-Kinase Regulatory Subunit 3
ANGPTL5: Angiopoietin-Like Protein 5
TEL: Translocation-Ets-Leukemia (Pathway)
ECM: Extracellular Matrix
IL-6: Interleukin-6
TNF-α: Tumor Necrosis Factor-Alpha
TGF-β: Transforming Growth Factor-Beta
Smad: Sma- and Mad-Related Protein (Pathway)
SNP: Single Nucleotide Polymorphism
GEO: Gene Expression Omnibus
DEG: Differentially Expressed Gene
FC: Fold Change
GO: Gene Ontology
BP: Biological Process
MF: Molecular Function
CC: Cellular Component
KEGG: Kyoto Encyclopedia of Genes and Genomes
PPI: Protein-Protein Interaction
SVM-RFE: Support Vector Machines with Recursive Feature Elimination
XGBoost: eXtreme Gradient Boosting
IV: Instrumental Variable
eQTL: Expression Quantitative Trait Locus
LD: Linkage Disequilibrium
OR: Odds Ratio
CI: Confidence Interval
TF: Transcription Factor
TFBS: Transcription Factor Binding Site
NCBI: National Center for Biotechnology Information
MsigDB: Molecular Signatures Database
NES: Normalized Enrichment Score
DGIdb: Drug-Gene Interaction Database
PCA: Principal Component Analysis
HPCA: Human Primary Cell Atlas
UMAP: Uniform Manifold Approximation and Projection
AUC: Area Under the Curve
VEC: Vascular Endothelial Cell
SMC: Smooth Muscle Cell
FIB: Fibroblast Cell
EMT: Epithelial–Mesenchymal Transition
ERAS: Enhanced Recovery After Surgery
PD-L1: Programmed Death-Ligand 1
FSCN1: Fascin Actin-Bundling Protein 1
MASP1: Mannan-Binding Lectin-Associated Serine Protease 1
PDK4: Pyruvate Dehydrogenase Kinase 4
FTH1: Ferritin Heavy Chain 1
SAT1: Spermidine/Spermine N1-Acetyltransferase 1
lncRNA: Long Non-Coding RNA
CASC11: Cancer Susceptibility Candidate 11
YBX1: Y-Box Binding Protein 1
HNRNPC: Heterogeneous Nuclear Ribonucleoprotein C
WDR77: WD Repeat Domain 77
circGLIS3: Circular RNA GLIS Family Zinc Finger 3
FTO: Fat Mass and Obesity-Associated Protein
EDN1: Endothelin 1
NTF3: Neurotrophin 3
