# Supplementary material for: Exploring hub genes related to adipocytokines in keloids: a combined analysis integrating single-cell, Mendelian randomization and bulk transcriptome data with experimental verification
Source: Front Mol Biosci. 2026 Mar 11;13:1740876. doi: 10.3389/fmolb.2026.1740876 (PMC13012983; doi:10.3389/fmolb.2026.1740876)
Supplement: Supplementary file 1 [file Supplementaryfile1.zip › Supplementary Table 17.docx]

**Supplementary** **Table 17** List of marker genes annotated for a single cell

| celltype | Marker_Gene |
| --- | --- |
| VEC | VWF、ENG、PECAM1、ACKR1、SELE |
| SMC | ACTA2、TAGLN、MYH11、MYL9 |
| FIB | COL1A1、DCN、COL1A2、COL3A1 |
| keratinocytes | HPGD |
| lymphatic endothelial cells | LYVE1 |
| schwann cell | NRXN1、S100B、SCN7A |
| melanocyte | MLANA、TYRP1、PMEL |
