# Supplementary material for: Exploring hub genes related to adipocytokines in keloids: a combined analysis integrating single-cell, Mendelian randomization and bulk transcriptome data with experimental verification
Source: Front Mol Biosci. 2026 Mar 11;13:1740876. doi: 10.3389/fmolb.2026.1740876 (PMC13012983; doi:10.3389/fmolb.2026.1740876)
Supplement: Supplementary file 1 [file Supplementaryfile1.zip › Supplementary Table 19.docx]

**Supplementary** **Table 19** List of marker genes annotated for a single cell

| celltype | Marker_Gene |
| --- | --- |
| FIB#1 | APOE、CXCL2、CXCL3 |
| FIB#2 | POSTN、ASPN、CPL1A2 |
| FIB#4 | CLD1 |
